# Anti-inflammatory and Regulatory Effects of Huanglian Jiedu Decoction on Lipid Homeostasis and the TLR4/MyD88 Signaling Pathway in LPS-Induced Zebrafish

**DOI:** 10.3389/fphys.2019.01241

**Published:** 2019-09-26

**Authors:** Junyi Zhou, Xinru Gu, Xiaorui Fan, Yanyan Zhou, Hongjie Wang, Nan Si, Jian Yang, Baolin Bian, Haiyu Zhao

**Affiliations:** Institute of Chinese Materia Medica, China Academy of Chinese Medical Sciences, Beijing, China

**Keywords:** Huanglian Jiedu decoction, anti-inflammatory activity, lipidomic profile, TLR4/MyD88 signaling pathway, lipopolysaccharide, zebrafish

## Abstract

Huanglian Jiedu decoction (HLJDD) has been used in the clinical treatment of inflammatory conditions. To clarify the mechanism of its comprehensive anti-inflammatory activities, the correlation between lipid homeostasis and the TLR4/MyD88 signaling pathway in zebrafish was established in the present study. In the lipopolysaccharide (LPS)-induced inflammation in zebrafish model, RT-PCR assays of five inflammatory cytokines and six targeted proteins were measured. Lipidomics analysis was conducted to identify potential lipid markers. HLJDD displayed strong efficacies, with a 61% anti-inflammatory rate at a concentration of 50 μg/mL. The activation of TLR4/MyD88 played an essential role in the inflammatory process. All protein indexes in the HLJDD group exhibited a tendency to reverse back to normal levels. Moreover, 79 potential pathological lipid biomarkers were identified. Compared with the model group, 61 therapeutic lipid biomarkers were detected in HLJDD group. Most perturbations of lipids were ameliorated by HLJDD, mainly through the glycerophospholipid metabolic pathway. In the visual network study, the corresponding lipoproteins such as PLA_2_, SGMS, and SMDP were observed as important intermediates between lipid homeostasis and the TLR4/MyD88 signaling pathway.

## Introduction

Inflammation is a complicated pathological process associated with many human diseases. It is a defense response of tissues against injury or damage (Coussens and Werb, 2002; Ryu et al., 2017). The persistence of cytokines and chemokines, as well as the triggered cascade reaction could induce tumor cell proliferation, promote inflammatory cell aggregation, and stimulate conditions such as lupus erythematosus, diabetes, cardiovascular diseases, and tumors (Wellen and Hotamisligil, 2005; Dominguez et al., 2017; Maffia and Cirino, 2017). Disorders of lipid homeostasis lead to the abnormal secretion of inflammatory mediators. Lipid oxidation and alterations in lipid structures are important triggers of inflammatory diseases, including atherosclerotic diseases, renal failure, and metabolic syndrome (Mi et al., 2012). Moreover, an exaggerated inflammatory response is able to affect intracellular lipid accumulation (Raetz et al., 2006; Colin et al., 2015). The processes of inflammation and lipid metabolism interact, and both are modulated through specific signaling pathways of NF-κB, MAPK, and PPAR (Nakaya et al., 2011; Ayaka et al., 2015; Gong et al., 2017).

TLR4 are key membrane recognition receptors for inflammation induced by bacterial lipopolysaccharide (LPS). The TLR4 receptors are capable of activating the transcription of inflammatory molecules via stimulation of the MyD88 pathway to directly regulate the release of pro-inflammatory cytokines or the Toll/IL-1(TIR) domain-containing adaptor inducing TRIF dependent pathway to produce interferon (Thada et al., 2013; Cai et al., 2015). The MyD88 pathway, and NF-κB and MAPK signaling pathways all comprehensively participate in the pathogenesis of inflammation and inflammatory responses. Among of them, p65, IκB-α belong to the NF-κB pathway. ERK, JNK, p38 belong to the MAPK pathway.

Zebrafish are highly homologous to mammals in terms of physiological signaling pathways and functions (Postlethwait et al., 2000). In recent years, zebrafish have been widely used to produce an *in vivo* model of LPS-stimulated inflammation because of their innate immune system comprising TLRs, neutrophils, and macrophages (Harvie et al., 2013; Hwang et al., 2016; Kim et al., 2018a; Ko et al., 2018). The number of neutrophils in zebrafish yolks is an important index by which the level of inflammation can be evaluated. Furthermore, the optical transparency of early developmental embryos facilitates the non-invasive and dynamic imaging of inflammation (D’Alençon et al., 2010). At present, the quantitative RT-PCR assay has become the main method of detection and examining the inflammatory factors in zebrafish models (Yang et al., 2014; Kim et al., 2018b).

HLJDD, which comprises Coptidis Rhizoma, Scutellariae Radix, Phellodendri Chinensis Cortex, and Gardeniae Fructus in the ratio 3:2:2:3, is a classical heat-clearing and detoxifying Chinese medicinal formula (Yang et al., 2013). It has been used in the treatment of inflammation-related diseases (Zhang et al., 2014; Wang et al., 2015). Furthermore, HLJDD could significantly reduce levels of inflammatory cytokines such as IL-2, TNF-α, IFN-γ, and inflammatory mediators such as prostaglandin E_2_ (PGE_2_) and NO to inhibit the immune response and inflammation (Wang and Xu, 2000; Fang et al., 2004). In our previous study, qualitative and quantitative analyses of HLJDD were performed using HPLC and LC/MS methods. Berberine, baicalin, and geniposide have been identified as three major components of HLJDD (Yang et al., 2013). They reduced inflammation in a dose-dependent manner by inhibiting cytokines through NF-κB and MAPK signaling pathways. The negative regulation of TLR4 is one important upstream mechanism of berberine, baicalin, and geniposide (Hong et al., 2012; Cui et al., 2014; Shi et al., 2014; Cheng et al., 2017; Wang et al., 2017; Zhang H. et al., 2017). In addition, the major constituents of HLJDD have also attracted increasing interest for their anti-inflammatory effects via the regulation of lipid homeostasis in fatty liver, hyperlipidemia, IPF, and other inflammation-related diseases (Gu, 2015; Hu et al., 2015; Chang et al., 2016; Meng et al., 2016; Zhang J. et al., 2017).

In the present study, HLJDD and three of its main active components exhibited significant anti-inflammatory effects in the zebrafish model of LPS-induced inflammation. Moreover, systematically non-targeted and targeted lipidomics analyses were performed to demonstrate the alterations in integrated lipid profiling after treatment with drugs. Furthermore, potential therapeutic lipid markers and their mainly metabolic pathways were identified by biofunctional analysis. Targeted proteins and cytokines regulated by the TLR4/MyD88 pathway were examined in all groups. Based on visualization and network analysis, the relationship between lipid markers and the TLR4/MyD88 signaling pathway was established via the significant intermediate bridge of corresponding lipoproteins. These findings would shed light on the clinical applications of HLJDD in inflammation-related disease.

## Materials and Methods

### Chemicals and Reagents

Huanglian Jiedu decoction (a traditional Chinese herb formula) was previously prepared in our laboratory. In addition, LPS, methyl cellulose, and ammonium acetate were purchased from Sigma-Aldrich Co., (St. Louis, MO, United States). Acetonitrile, methanol, and formic acid of mass spectrometry (MS) grade were obtained from Fisher Scientific (Fair Lawn, NJ, United States). Trizol and ddH2O (DNase/RNase Free) were purchased from Ambion (United States); HiScript Reverse Transcriptase (RNase H), 5×HiScript Buffer, 50 × ROX Reference Dye 2, and SYBR Green Master Mix were purchased from VAZYME (China). Ribonuclease Inhibitor was purchased from *TRANS* (China); dNTP, Taq Plus DNA Polymerase, and DL2000 DNA Marker were purchased from TIANGEN (China); and Random Primer (N6) were purchased from AIDLAB (China). The standards of berberine, baicalin, and geniposide were obtained from Saibaicao Technology Co., Ltd., (Beijing, China). Indomethacin was purchased from Shanghai Jingchun Industrial Co., Ltd., (Shanghai, China). The purities of all standard references were over 98%. All other chemicals and solvents were of analytical grade.

### Animal Maintenance

Zebrafish were maintained at a temperature of 28°C with a 14:10 h (day/night) cycle and fed three times per day. The quality of the rearing water was 200 mg of instant sea salt per liter of reverse osmosis water, the conductivity was 480∼510 μS/cm, the hardness was 53.7∼71.6 mg/L CaCO_3_, and the pH was 6.9∼7.2. Transgenic zebrafish exhibiting neutrophil fluorescence (MPO-GFP) were purchased from Hunter Biotechnology, Inc., (Hangzhou, China). The MPO-GFP transgenic zebrafish line was selected for this study and all animal experiments were performed in accordance with the guidelines issued by the Animal Ethics Committee (Association for Assessment and Accreditation of Laboratory Animal Care International (AAALAC) Certificate NO.001458).

### Establishment of Inflammation Model and Drug Administration

The yolks of zebrafish larva were injected with 10 mg/mL LPS at 3 days post-fertilization (dpf). A volume of 10 nL was administered to each embryo to establish the inflammatory model. Indomethacin was used as the positive control (28.6 μg/mL). Based on the maximum solubility of the substances and the tolerance of zebrafish in the maximum concentration, the MTC of HLJDD, berberine, baicalin, and geniposide in zebrafish were determined at first. The data were shown in Supplementary Table S1. Then in the *in vivo* susceptibility test, five concentrations of MTC/16, MTC/8, MTC/4, MTC/2, and MTC were set for each drug. Three hours after administration of the treatments, 10 transgenic zebrafish (MPO-GFP) larvae were randomly selected from each experimental group to monitor the inflammatory neutrophil migration process in real time under a fluorescence microscope. Image analysis was performed using the Nikon NIS-Elements D 3.10 advanced image processing software to calculate the number of fluorescent neutrophils (N), and quantitatively evaluate the inhibitory effect of each anti-inflammatory drug on neutrophils in zebrafish. The formula for calculating the inhibition of inflammation was as follows:

Extinctioneffectofinflammation(%)=[N(Drugtreatmentgroup)-N(Controlgroup)]/N(Drugtreatmentgroup)×100%

### RNA Extraction and Quantitative Real-Time PCR

Zebrafish larvae at 3 dpf (n = 20) were collected for total RNA extraction, frozen in 1.5 mL micro-centrifuge tubes using liquid nitrogen, and stored at −80°C. Total RNA was isolated using the Trizol reagent (Ambion, United States), and 2 μg aliquots of total RNA were reversely transcribed into cDNA for real-time quantitative PCR using a cDNA Kit. Real-time quantitative PCR was performed with the SYBR Green Master Mix, which was used to determine the expression levels of all genes. The protocol for the real-time PCR was as follows: 50°C for 2 min; 95°C for 10 min; 40 cycles at 95°C for 30 s; followed by 60°C for 30 s. The 2^−△△*Ct*^ method was used to calculate the relative expression of each gene using β-actin for normalization. The experiments were repeated three times. The primer sequences used in the present study are shown in Table 1.

**TABLE 1:** Primer sequences for the RT-PCR assay.

**Gene symbol**	**Oligo**	**Primer sequence**	**Size (bp)**
β-actin	Forward	5′- CCATCTATGAGGGTTACGC -3′	137
	Reverse	5′- GACAATTTCTCTTTCGGCT -3′	
TNF-α	Forward	5′- GCTGGATCTTCAAAGTCGGGTGTA -3′	139
	Reverse	5′- TGTGAGTCTCAGCACACTTCCATC -3′	
IL-1β	Forward	5′- CTCAGCCTGTGTGTTTGGGA -3′	209
	Reverse	5′- GGGACATTTGACGGACTCG -3′	
IL-6	Forward	5′- ACGACATCAAACACAGCACC -3′	172
	Reverse	5′- TCGATCATCACGCTGGAGAA -3′	
MyD88	Forward	5′- ACCATCGCCAGTGAGCTTAT -3′	206
	Reverse	5′- CAGATGGTCAGAAAGCGCAG -3′	
ERK	Forward	5′- TCCAAGGGCTACACCAAGTC -3′	274
	Reverse	5′- TGCGATCCAATAAATCCAAA -3′	
JNK	Forward	5′- ATTGCCTTTTGTCAGGGTTT -3′	140
	Reverse	5′- TACCGTTTGAGAACCGTGAA -3′	
p38	Forward	5′- GTCGCAGAAAGAAAGACCCA -3′	130
	Reverse	5′- ATCAAACGCAGAGCAAACAG -3′	
IκB-α	Forward	5′- GTTGGATTCGTTAAAAGAGGA -3′	137
	Reverse	5′- GGATAATGGCGAGATGTAGAT -3′	
p65	Forward	5′- CGCAAGAGAACTGAAGGAA -3′	205
	Reverse	5′- AGAAAAAGGAGGTGGGTGG -3′	
IL-10	Forward	5′- GGAGACCATTCTGCCAACA -3′	111
	Reverse	5′- CATTTCACCATATCCCGCT -3′	
IFN-γ	Forward	5′- GTTTGCTGTTTTCGGGATGG -3′	138
	Reverse	5′- TTCGCAGGAAGATGGGGTGT -3′	

### Sample Pretreatment

Zebrafish samples were thawed at room temperature. Thereafter, 1 mL of methanol/chloroform (1:3) organic solvent was added to a 1.5 mL Eppendorf tube containing each zebrafish sample. The samples were then sonicated for 10 min and vortexed for 50 min. A volume of 100 μL of water was added to each sample, following which it was centrifuged at 12 000 rpm and 4°C for 10 min. A volume of 500 μL of the subnatant was collected and snapped-frozen in liquid nitrogen, after which 200 μL of isopropanol/acetonitrile (1:1) was added for reconstitution. The samples were then sonicated and centrifuged at 12 000 rpm and 4°C for 10 min. The supernatant was then analyzed by ultra-high performance liquid chromatography coupled to Q Exactive hybrid quadrupole-Orbitrap mass spectrometry.

### UPLC/Q-Exactive/MS Analysis

The UltiMate^TM^ 3000 Rapid Separation LC (RSLC) system (Thermo Scientific, United States) was used for the relative quantification of different kinds of lipids. The Waters Acquity UPLC HSS T3 column (2.1 × 100 mm, 1.8 μm) was used for the separation of lipids; acetonitrile/water (60/40) was used for mobile phase A and isopropanol/acetonitrile (90/10) was used for mobile phase B. Both A and B phases contained 0.1% formic acid and 10 mmol/L ammonium acetate. The samples were eluted using the following linear gradient conditions: 0∼2 min, 20∼30% B; 2∼5 min, 30∼45% B; 5∼6.5 min, 45∼55% B; 6.5∼12 min, 55∼65% B; 12∼14 min, 65∼85% B; 14∼17.5 min, 85∼100% B; 17.5∼18 min, 100∼100% B, and the equilibration time was 1.5 min with 20% B. The column was operated at 45°C and the flow rate was 300 μL/min.

A Thermo Scientific^TM^ Q Exactive hybrid quadrupole-Orbitrap mass spectrometer equipped with a HESI-II probe was employed in both positive and negative ionization modes. The HESI-II spray voltages were 3.7 kV and −3.5 kV, respectively. The heated capillary temperature was 320°C, the sheath gas pressure was 30 psi, the auxiliary gas setting was 10 psi, and the heated vaporizer temperature was 300°C. Nitrogen was used as both the sheath gas and the auxiliary gas. Nitrogen was also used as the collision gas at a pressure of 1.5 mTorr. The parameters of the full mass scan were as follows: resolution of 70,000, auto gain control target under 1.0 × 10^6^, maximum isolation time of 50 ms, and m/z range of 50–1500 Da.

### Data Processing and Statistical Analysis

All MS data in both positive and negative ion modes were processed using the Skyline software^1^ for targeted lipidomics, and the Progenesis QI software (Non-linear Dynamics, Newcastle, United Kingdom) for untargeted lipidomics by imputing raw data, peak alignment, peak picking, and normalization to produce peak intensities for the retention time (t_*R*_), m/z data pairs, and abundance. The automatic peak picking ranged from 1 to 19 min.

To monitor the stability and reproducibility of the system in each batch under analysis, QC samples were prepared by mixing equal volumes of each zebrafish sample. Three QC samples were continuously injected at the beginning of the run. Six QC samples were then injected at regular intervals throughout the analytical run in order to provide data from which repeatability could be assessed. Preprocessed data were imported to the SIMCA 14.1 software (Umetrics AB, Umea, Sweden) and EZinfo 2.0 software (Waters Corporation, Manchester, United Kingdom) for multivariate analysis. Unsupervised separation was assessed by PCA using Parteo standardization to assess the differences in the lipids of zebrafish samples among all variables. The data were further processed by OPLS-DA to identify the potential lipid markers based on the VIP values (VIP > 1), *t*-test (*P* < 0.05), and fold change (FC > 1.3 or FC < 0.7). The external model was verified by the permutation test to evaluate the risk of over-fitting. The HMDB database^2^, and the LIPID MAPS^3^ and MetaboAnalyst^4^ tools were used to identify the selected potential lipid structures through multistage mass spectra information fragments and analyze the possible metabolic pathway influenced by HLJDD. R language software (x64 3.5.2) was used to install the packages (“Venn Diagram”) and (“corrplot”) for the classification and assignment of the lipid biomarkers in each group and correlation analysis. Cytoscape v3.7.1 software was used for network visualization of differentially expressed lipid marker species and the TLR4/MyD88 signaling pathway via the corresponding lipoproteins by setting the value of degree, closeness and betweenness.

All data were presented as mean ± SD. Significant differences between the various groups were determined by one-way ANOVA, followed by LSD *post hoc* tests, using GraphPad prism version 7.01. In all cases, a value of *P* < 0.05 was considered significant.

## Results and Discussion

### Anti-inflammatory Effects of HLJDD, Berberine, Baicalin, and Geniposide in LPS-Stimulated Zebrafish

Compared with the control group, the number of neutrophils in the LPS model group was significantly increased (*P* < 0.001). The MTCs of HLJDD, berberine, baicalin, and geniposide in LPS-induced zebrafish were 100, 62.5, 250, and 500 μg/mL, respectively. Five concentrations were selected based on the MTC to evaluate the dose-effect relationships of HLJDD (6.25, 12.5, 25, 50, and 100 μg/mL); berberine (3.9, 7.8, 15.6, 31.25, and 62.5 μg/mL); baicalin (15.6, 31.25, 62.5, 125, and 250 μg/mL); and geniposide (31.25, 62.5, 125, 250, and 500 μg/mL).

**FIGURE 1:**
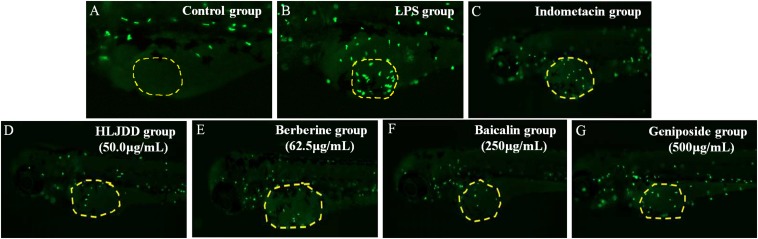
Representative pictures of transgenic neutrophils labeling in zebrafish yolks at 3 dpf. **(A)** Control group. **(B)** Lipopolysaccharide (LPS) group. **(C)** Indomethacin group. **(D)** 50.0 μg/mL Huanglian Jiedu decoction (HLJDD) group. **(E)** 62.5 μg/mL Berberine group. **(F)** 250 μg/mL Baicalin group. **(G)** 500 μg/mL Geniposide group. Green fluorescence represents the transgenic neutrophils.

The results demonstrated that HLJDD had the strongest efficacy at a concentration of 50 μg/mL with 61% anti-inflammatory activity among four drugs. Moreover, the efficacies of berberine, baicalin, and geniposide were distinctly exhibited in a concentration-dependent manner. The most favorable effects of HLJDD, berberine, baicalin, and geniposide were exhibited at concentrations of 50, 62.5, 250, and 500 μg/mL. Their anti-inflammatory activities were 61, 61, 56, and 33%, respectively. The anti-inflammatory activity of indomethacin was 33% at a concentration of 28.6 μg/mL (Figure 1 and Table 2).

**TABLE 2:** Quantitative results of the effects among each experimental group demonstrating zebrafish inflammation (*n* = 10).

**Group**	**Concentration (μg/mL)**	**Number of neutrophils (mean ± SE)**	**Anti-inflammatory effects (%)**
Control	–	2 ± 0.29	–
LPS	–	18 ± 1.43	–
Indomethacin	28.6	12 ± 1.00^∗∗∗^	33^∗∗∗^
HLJDD	6.25	14 ± 1.53	22
	12.5	11 ± 1.13^∗∗∗^	39^∗∗∗^
	25	8 ± 0.96^∗∗∗^	56^∗∗∗^
	50	7 ± 0.95^∗∗∗^	61^∗∗∗^
	100	10 ± 1.58^∗∗∗^	44^∗∗∗^
Berberine	3.9	16 ± 1.64	11
	7.8	14 ± 1.00^∗^	22^∗^
	15.6	12 ± 0.73^∗∗∗^	33^∗∗∗^
	31.25	9 ± 1.05^∗∗∗^	50^∗∗∗^
	62.5	7 ± 0.88^∗∗∗^	61^∗∗∗^
Baicalin	15.6	17 ± 1.07	6
	31.25	13 ± 0.40^∗∗∗^	28^∗∗∗^
	62.5	10 ± 0.90^∗∗∗^	44^∗∗∗^
	125	9 ± 0.73^∗∗∗^	50^∗∗∗^
	250	8 ± 0.54^∗∗∗^	56^∗∗∗^
Geniposide	31.25	18 ± 1.34	0
	62.5	15 ± 1.23	17
	125	14 ± 0.70	22
	250	13 ± 1.62^∗^	28^∗^
	500	12 ± 0.96^∗∗^	33^∗∗^

*^∗^*P* < 0.05, ^∗∗^*P* < 0.01, and ^∗∗∗^*P* < 0.001, drug treatment group (or positive group) compared with LPS group.*

### The mRNA Expression of Six Target Proteins and Five Inflammatory Cytokines

**FIGURE 2:**
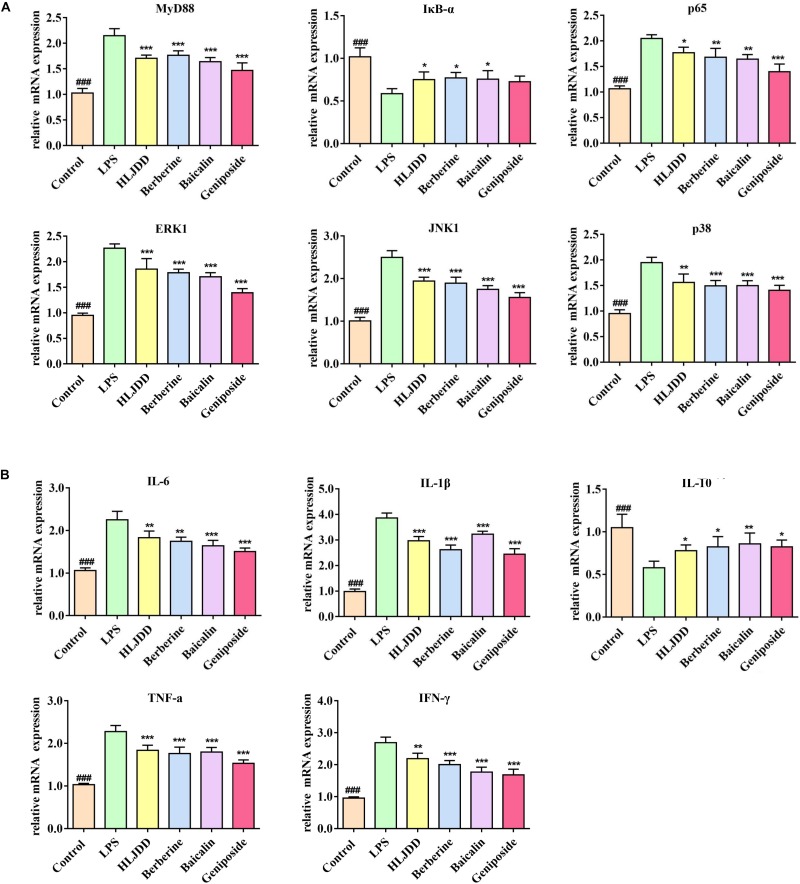
Huanglian Jiedu decoction (HLJDD) and its three major components alleviated the mRNA expression of the key mediators and inflammatory cytokines. **(A)** HLJDD and its three major components could inhibit lipopolysaccharide (LPS)-induced inflammation by ameliorating the mRNA expression of MyD88, p65, IκB-α, ERK, JNK, and p38 controlled by the TLR4/MyD88 pathway. **(B)** HLJDD and its three major components ameliorated IL-6, IL-1β, TNF-α, IL-10, and IFN-γ to return to normal level. ^∗^*P* < 0.05, ^∗∗^*P* < 0.01, and ^∗∗∗^*P* < 0.001, four drug treatment groups compared to the LPS group. ^###^*P* < 0.001, LPS group compared to the control group. MyD88: Binding Toll-like Receptor. p65: Response to cytokine. IκB-α: Negative regulation of NF-κB transcription factor activity. ERK, JNK, p38: Activation of MAPK activity. IL-6, IL-1β, TNF-α, IL-10, IFN-γ: Regulation of inflammatory response.

In the LPS group, the levels of IκB-α were significantly decreased by 40%, whereas those of p65, ERK, JNK, and p38 were increased by approximately 1–2 fold greater compared with the control group. Furthermore, IL-6, IL-1β, TNF-α, and IFN-γ were significantly induced, whereas IL-10 was reduced by the LPS stimulus. The expression of IL-1β in the LPS group showed the greatest variation, which was approximately 4-fold greater than that in the control group. All of the aforementioned eleven indexes presented a tendency to normal levels in all of the four drug treatment groups, especially in the geniposide group. Compared with the LPS group, berberine induced a notable increase in IκB-α levels by 30%, and IL-10 levels by 40%, and a reduction in IL-1β levels by about 30%. Baicalin significantly increased IκB-α levels by 30% and IL-10 levels by 50%, and simultaneously reduced JNK levels by 30% and IFN-γ levels about 30% (Figures 2A,B). Furthermore, MyD88, as the first responsive intracellular signaling molecule, played a dominant role in its association with inflammatory cytokines (O’Neill and Bowie, 2007). The phosphorylation and degradation of the IκB-α protein led to its dissociation from NF-κB, which promoted the transfer of NF-κB into the nucleus to participate in the regulation of the expression of inflammatory cytokines (Sun and Andersson, 2002). Based on the RT-PCR results of six target proteins and five cytokines, HLJDD, berberine, baicalin, and geniposide displayed similar anti-inflammatory properties in response to LPS stimulus, which was consistent with the results of previous studies (Hong et al., 2012; Cui et al., 2014; Shi et al., 2014; Wang et al., 2017; Zhang H. et al., 2017). The results all prove that the regulation of NF-κB and MAPK signaling pathways play important roles via the anti-inflammatory mechanisms.

**FIGURE 3:**
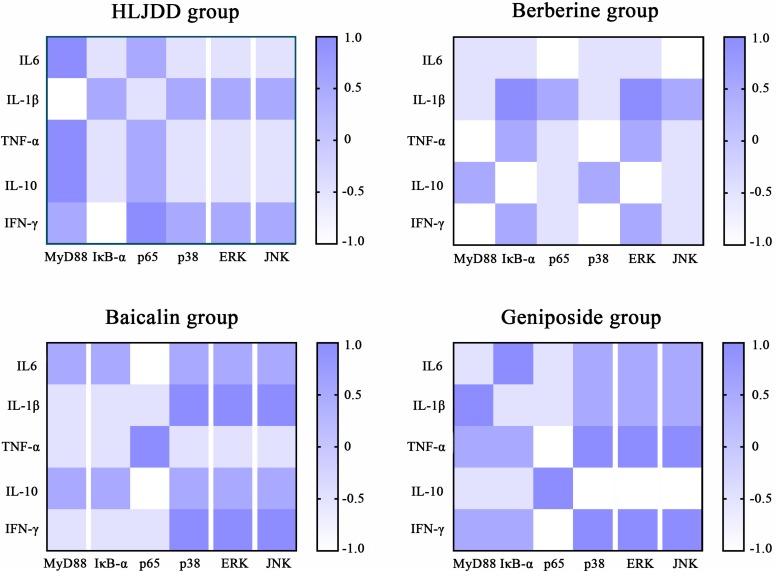
Correlation analysis of inflammatory cytokines and mediators of the TLR4/MyD88 signaling pathway in each drug treatment group. The color scale illustrates the magnitude of correlation between the examined indexes on the plot.

Based on the correlation analysis of the four drug treatment groups (Figure 3), the inflammatory cytokines and mediators of the TLR4/MyD88 pathway showed different degrees of correlation. In the HLJDD group, MyD88 was closely associated with IL-6, TNF-a, IL-1β, and IL-10. In the baicalin group, the secretion of IL-1β and IFN-γ via ERK, JNK, and p38 exhibited significant differences with correlation coefficients at 0.5 and 1.0, respectively. In addition, these cascade kinases and p65 were closely associated with the secretion of TNF-a, IL-10, and IFN-γ in the geniposide group.

### Identification of Non-targeted Lipid Profiling Data of Zebrafish

**FIGURE 4:**
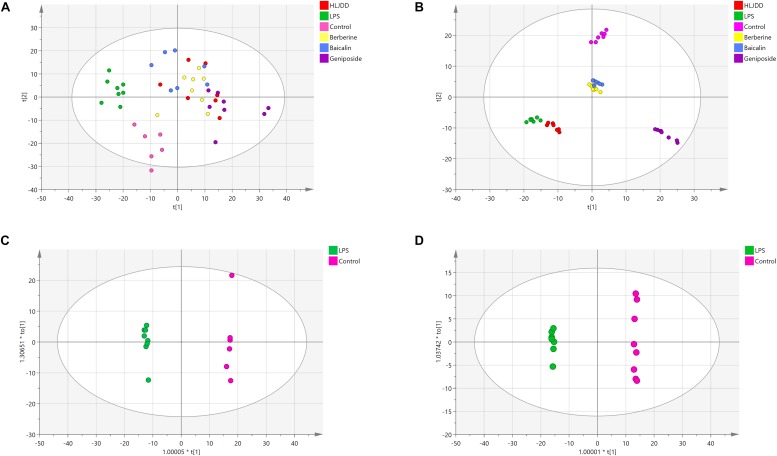
Principal component analysis (PCA) scores of zebrafish samples in six groups and orthogonal projection to latent structures discriminant analysis (OPLS-DA) scores in the control group and lipopolysaccharide (LPS) group. **(A)** PCA analysis in ESI^+^ mode. **(B)** PCA analysis in ESI^–^ mode. **(C)** OPLS-DA analysis in ESI^+^ mode. **(D)** OPLS-DA analysis in ESI^–^ mode. Red, green, pink, yellow, blue, and purple symbols represent samples of the HLJDD, LPS, control, berberine, baicalin, and geniposide groups, respectively.

Based on the chromatographic retention time, accurate masses, and MS/MS fragments, 2384 positive ion peaks and 725 negative ion peaks were obtained. The plot of PCA scores exhibited distinct clusters of zebrafish in six groups. The cumulative *R*^2^*X* and *Q*^2^ were 0.824 and 0.666, respectively, in the ESI^+^ mode, and 0.792 and 0.708, respectively, in the ESI^–^ mode (Figures 4A,B). In the plot of OPLS-DA scores, a clear separation was noted between the control group and the LPS group. The cumulative *R*^2^*Y* and *Q*^2^ were 0.998 and 0.903, respectively, in the ESI^+^ mode, and 0.999 and 0.996, respectively, in the ESI^–^ mode (Figures 4C,D). According to the permutation test, no over-fitting tendency was evident, which indicated that the LPS-induced zebrafish inflammatory model had a favorable predictive ability for the screening of potential lipid markers. A volcano plot showed all indexes under examination. The dots indicating high ejection were considered to be very significant (Supplementary Figure S1). The levels of potential lipid markers in the LPS and drug treatment groups were screened and identified by untargeted lipidomics analysis and are presented in Supplementary Table S2. The potential lipid markers were mainly phosphatidylcholines (PCs) and phosphatidylethanolamines (PEs). The results of previous pilot experiments also support the data (Zhou et al., 2018).

### Identification of Targeted Lipid Profiling Data of Zebrafish

Based on the non-targeted lipidomics results, we focused on PCs, PEs, sphingomyelins (SMs), ceramides (Cers), and triglycerides (TGs) for the analysis of targeted lipidomics. The database covered 97 PCs, 45 Cers, 27 PEs, and 24 SMs. The plot of PCA 3D scores exhibited distinct clusters of six zebrafish groups (Supplementary Figure S2).

**FIGURE 5:**
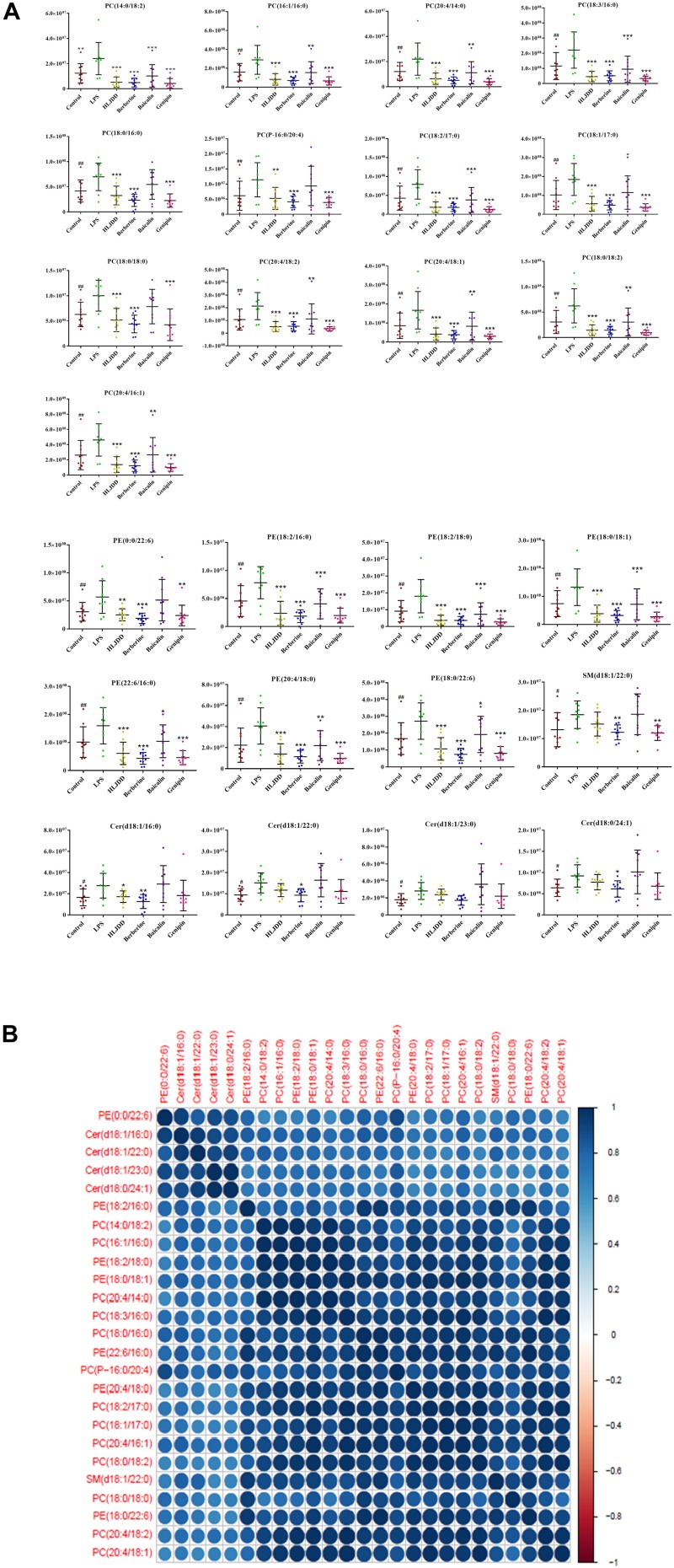
Multivariate statistical analysis of the lipopolysaccharide (LPS) group and control group. **(A)** The metabolic changes of 25 potential lipid markers in LPS group. ^∗^*P* < 0.05, ^∗∗^*P* < 0.01, and ^∗∗∗^*P* < 0.001, four drug treatment groups compared to the LPS group. ^#^*P* < 0.05 and ^##^*P* < 0.01, LPS group compared to the control group. **(B)** Relationship of correlation matrix between clusters of 25 potential lipid markers. Different color scales reflect the magnitude and direction of correlation between different lipids. Blue and red indicate positive and negative correlations, respectively.

A total of 25 pathological potential lipid markers were further discovered following induction by LPS, including 13 PCs, seven PEs, four Cers, and one SM. Specifically, PC(14:0/18:2) and PC(20:4/16:1) exhibited the highest expression in 25 biomarkers in the LPS group. After the administration of HLJDD, lipid levels of 28 PCs and eight PEs were significantly identified and exhibited a tendency to normal levels. The increase in the levels of 28 PCs and 11 PEs was reversed by the berberine treatment. In the baicalin group, 14 PCs and four PEs were identified. In the geniposide group, 33 PCs, nine PEs, and one SM were observed to have a tendency to normal levels. The identification of potential lipid markers are presented in Figure 5. The significant associations among 25 markers were assessed by correlation analysis. From a holistic view, all 25 markers were strongly correlated with each other, which supports the underlying explanation for the pathogenesis of inflammation induced by LPS. The levels of potential lipid markers in the four drug treatment groups analyzed by targeted lipidomics are shown in Supplementary Table S3.

### Metabolic Pathway Analysis of HLJDD Treatment

**FIGURE 6:**
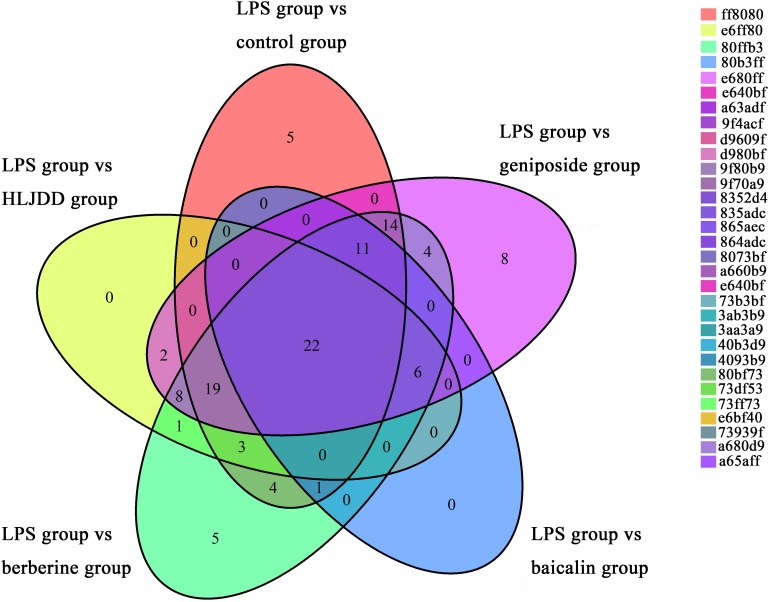
Venn diagram, in which each ellipse represents the potential lipid markers based on comparisons of the lipopolysaccharide (LPS) group *vs.* drug treatment groups and LPS group *vs.* the control group.

**FIGURE 7:**
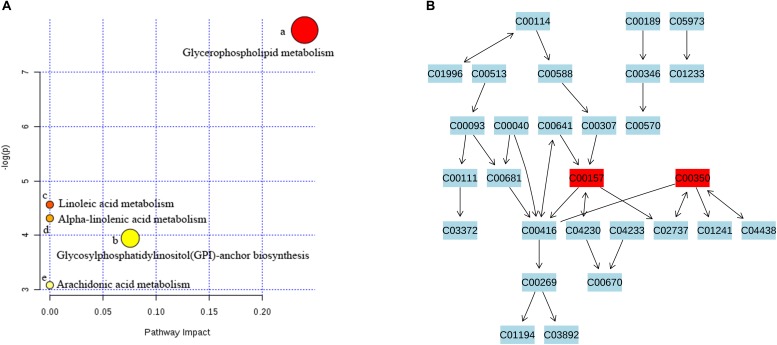
Metabolic pathway analysis and visualization of Huanglian Jiedu decoction (HLJDD) treatment. **(A)** Summary of metabolomics pathway analysis based on the KEGG database. All matched pathways are presented as circles, including glycerophospholipid metabolism (a); glycosylphosphatidylinositol (GPI)-anchor biosynthesis (b); linoleic acid metabolism (c); alpha-linolenic acid metabolism (d); and arachidonic acid (AA) metabolism (e) from significantly differential lipids. The color and size of each circle are based on *P*-value and pathway impact value, respectively. **(B)** Pathway of glycerophospholipid metabolism with metabolomics pathway analysis based on the KEGG database. The matched metabolite numbers are consistent with the KEGG database.

As shown in the Venn diagram (Figure 6), 22 common potential lipid markers were identified based on both targeted and non-targeted lipidomics in each group. In addition, berberine ameliorated five lipid markers, including three TGs, one PS, and one PE. Geniposide simultaneously ameliorated eight lipid markers of seven PCs and one SM. Furthermore, potential metabolic pathways influenced by HLJDD treatment were investigated for further study. Among the five important pathways under observation, glycerophospholipid metabolism was deemed the most vital metabolic pathway with the highest impact value of 0.240 (Figure 7A). The detailed construction of crucial metabolites in the glycerophospholipid pathway based on the KEGG database is shown in Figure 7B.

### Network Visualization Analysis of “Lipid Markers-Lipoproteins-TLR4/MyD88 Pathways” for the Anti-inflammatory Effects of HLJDD

**FIGURE 8:**
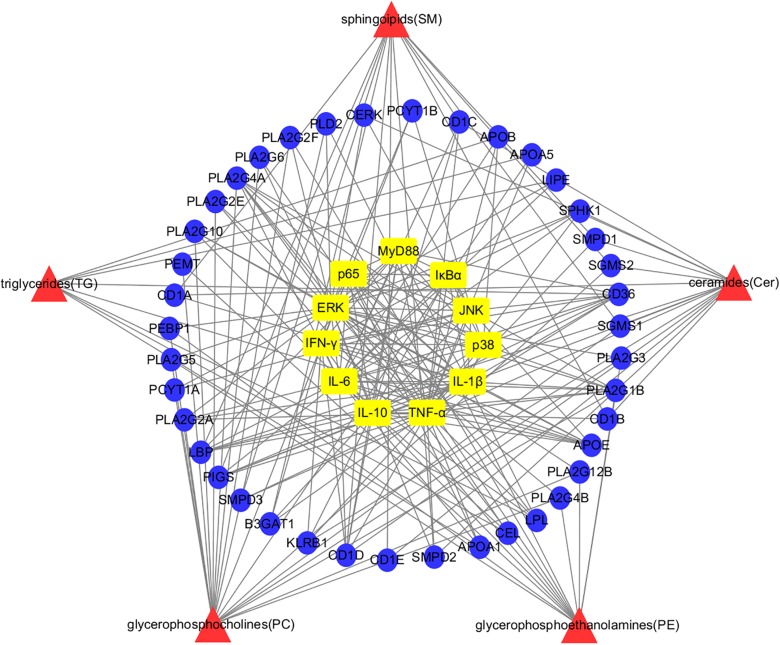
Network visualization analysis of “potential lipid markers-lipoproteins-TLR4/MyD88 signaling pathways” for anti-inflammatory effects of HLJDD. Red area: differentially expressed species of lipid markers; Blue area: corresponding lipoproteins of lipid markers; Yellow area: cytokines and proteins of the TLR4/MyD88 signaling pathways.

Based on the HMDB database, 40 corresponding lipoproteins were retrieved according to the matching lipid markers. Correlations between differentially expressed lipid marker species and the lipoproteins associated with the TLR4/MyD88 signaling pathway was then established using network visualization analysis (Figure 8). The edges of the nodes in the analysis directly or indirectly interacted with each other in varying degrees. A series of PLA_2_ lipoproteins were predominantly associated with PCs and PEs. Furthermore, PEMT, APO, PEBP1, and PLD2, among others were also involved in the synthesis of PCs and PEs. The SMs and Cers interacted with SGMS, SMPD, and CD1, among others. Moreover, CD36, CEL, LPL, LIPE, and a variety of apolipoproteins (APO) were identified as the regulatory lipoproteins linking TGs to the TLR4/MyD88 signaling pathway.

The PCs and PEs accounted for a large proportion of the total number of potential lipid markers in this study. Most were polyunsaturated subclasses containing 20:4, 18:1, 18:2, and 16:1 carbon chains. After appropriate stimulation, the glycerophospholipid content, containing 16-C and 18-C fatty acid chains, was increased, which is consistent with the results of a previous study (Rouzer et al., 2007). Moreover, in the lipid metabolic network, glycerophospholipids containing a 20:4 carbon chain are important precursors of a wide range of bioactive lipid mediators, such as prostaglandins, leukotrienes, and lipoxins, among others (Mason et al., 1972; Humes et al., 1977; Scott et al., 1980; Rouzer et al., 1982; Abiko et al., 1983). The PLA_2_s are limited enzymes produced by AA, PGE_2_, and other bioactive substances, which play important roles in the occurrence, development, and prognosis of inflammatory diseases (Yang and Xia, 2006). cPLA_2_ is produced by AA, which is from the glycerophospholipids of membranes (Murakami et al., 2011; Cao et al., 2013). In addition, owing to an up-regulation of CTL1 gene transcription, LPS induced a sharp increase in PC synthesis, which affected the acute response to LPS stimulation (Snider et al., 2018). The uptake of choline was possibly restricted after administration of HLJDD, which caused the reduction in the levels of PC and alterations in cytokine secretion. The cPLA_2_s were able to regulate the PKC∼ζ as well, which improved the nuclear translocation of NF-κB and enhanced the transcription of κB-dependent vectors (Arenzana-Seisdedos et al., 1993). Moreover, the dependence of PC-PLC on LPS-mediated activation of PKC-ζ is also associated with the activation of MAPK ERK 1/2 (Monick et al., 1999), which is a downstream signal of PKC (Lin and Zhang, 1996).

The levels of SMs and Cers were regulated by SGMS and SMPD in the SM cycle. Overexpression of SGMS_1_ and SGMS_2_ enhanced apoptosis mediated by the activation of DAG and PKC. As a neutral hydrolase, SMPD_3_ inhibited both proliferation and the cell cycle via SM hydrolysis, as well as the succeeding increase in the levels of Cers. The lack of SGMS_2_ resulted in the degradation of NF-κB and the expression of related genes (Hailemariam et al., 2008; Libby, 2012). The CD_1_ family comprises crucial structures that are required for interactions between accessory cells and T cells. Furthermore, SM is bound to the CD1D isoform. The CD-I type participates in the regulation of inflammation (Cox et al., 2009).

The main source of Cers is SMs, which are involved in a variety of important cellular signaling pathways and physiological processes (Hannun and Obeid, 2008). The Cers are considered important second signal effector molecules (Cuschieri, 2006). They activate the NF-κB and MAPK/JNK cascade signaling pathways, and up-regulate transcription factors associated with inflammation (Brenner et al., 1997; Shirakabe et al., 1997; Bourbon et al., 2000). The Cers are also hydrolyzed to sphingosine and then phosphorylated to S1P by SphK1 (Maceyka and Spiegel, 2014). All of these enzymes could regulate the activation of TLR4 stimulated by LPS (Vallabhapurapu and Karin, 2009).

The metabolism of TGs has a strong correlation with inflammation. The transport of TGs in lipoproteins is facilitated by polar lipids comprising phospholipids, a variety of APOs and unesterified cholesterol (Welty, 2013). As a co-receptor for TLRs in the recognition of pathogen-associated lipids, CD36 contributes to inflammatory resolution (Stuart et al., 2005; Ballesteros et al., 2014). One of its physiological functions is the high-affinity uptake of long-chain FAs from TG-rich lipoproteins. Furthermore, CD36 affects the remodeling of myocardial phospholipids and eicosanoid production. In cases of excessive FA supply, CD36 leads to lipid accumulation, dysfunction, and inflammation (Abumrad and Goldberg, 2016).

## Conclusion

In the present study, a total of 79 aberrantly changed lipid markers were confirmed in the LPS model group. Furthermore, 61 lipid markers were differentially expressed in the HLJDD treatment group, most of which were associated with PCs, PEs, SMs, Cers, and TGs. HLJDD played an important role in attenuating LPS-induced inflammation.

The visualization network correlation analysis of lipid biomarkers and the TLR4/MyD88 signaling pathways demonstrated that lipid homeostasis is a significant anti-inflammatory mechanism of HLJDD. In addition, lipoproteins were found to be a significant intermediate bridge between lipid metabolism and inflammatory pathways. Further research should be conducted to validate the role of lipoproteins and their association with the anti-inflammatory effects of HLJDD.

## Data Availability Statement

All datasets generated for this study are included in the manuscript/Supplementary Files.

## Author Contributions

JZ wrote the manuscript and performed the experiments. XG, XF, and YZ performed the experiments. HW, NS, and JY analyzed the data. BB and HZ designed the experiments. All authors reviewed the final manuscript.

## Conflict of Interest

The authors declare that the research was conducted in the absence of any commercial or financial relationships that could be construed as a potential conflict of interest.

## Footnotes

1 http://skyline.gs.washington.edu/

2 http://www.hmdb.ca/

3 http://www.lipidmaps.org/

4 http://www.metaboanalyst.ca/

## Abbreviations

AA: arachidonic acid
APO: apolipoproteins
cDNA: complementary DNA
Cers: ceramides
cPLA2: cytosolic PLA2
CTL1: choline transporter like protein
dpf: days post fertilization
ERK: extracellular signal regulated kinase
FAs: fatty acids
FC: fold change
GPI: glycosylphosphatidylinositol
HLJDD: Huanglian Jiedu decoction
I κ B- α: NF- κ B inhibitor- α
IFN- γ: interferons- γ
IL-1 β: interleukin-1 β
IL-10: interleukin-10
IL-6: interleukin-6
IPF: idiopathic pulmonary fibrosis
JNK: c-JunN-terminal kinase
KEGG: Kyoto Encyclopedia of Genes and Genomes
LPS: lipopolysaccharide
LSD: least significant difference
MAPK: mitogen protein kinase
MAPK: mitogen-activated protein kinase
MTC: maximum tolerated concentration
MyD88: myeloid differentiation primary response gene 88
NF- κ B: nuclear factor- κ B
NO: nitric oxide
OPLS-DA: orthogonal projection to latent structures discriminant analysis
PA: phosphatidic acid
PCA: principal component analysis
PC-PLC: PC-specific phospholipase C
PCs: phosphatidylcholines
PEs: phosphatidylethanolamines
PGE2: prostaglandin E2
PKC ∼ζ: zeta isoform of protein kinase C
PPAR: peroxisome proliferator-activated receptor
PSs: phosphatidylserines
QC: quality control
RT-PCR: reverse transcription polymerase chain reaction
S1P: sphingosine-1 phosphate
SD: standard deviation
SMs: sphingomyelins
SphK1: sphingosine kinases 1
TGs: triglyceride
TIR: Toll/IL-1
TLR4: toll like receptors 4
TNF- α: tumor necrosis factor- α
tR: retention time
VIP: variable importance in the projection
