# Supplementary material for: Anti-inflammatory and Regulatory Effects of Huanglian Jiedu Decoction on Lipid Homeostasis and the TLR4/MyD88 Signaling Pathway in LPS-Induced Zebrafish
Source: Front Physiol. 2019 Sep 26;10:1241. doi: 10.3389/fphys.2019.01241 (PMC6775191; doi:10.3389/fphys.2019.01241)
Supplement: Supplementary file 1 [file Data_Sheet_1.PDF]

Supplementary Material

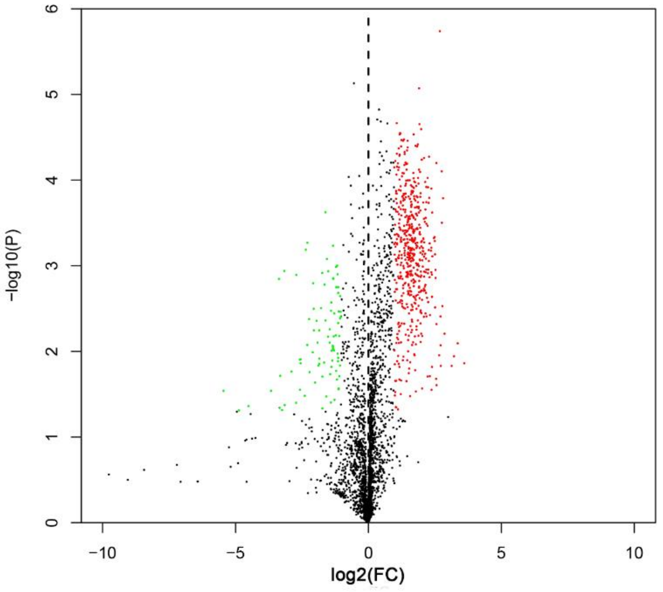

Figure S1. Volcano plot of the potential lipid markers in LPS group vs control group based on the indexes of *P* and FC. The black dots represented all the examined indexs. The red dots represented the up-regulated lipids, while the green dots represented the down-regulated lipids in LPS group compared with the control group.

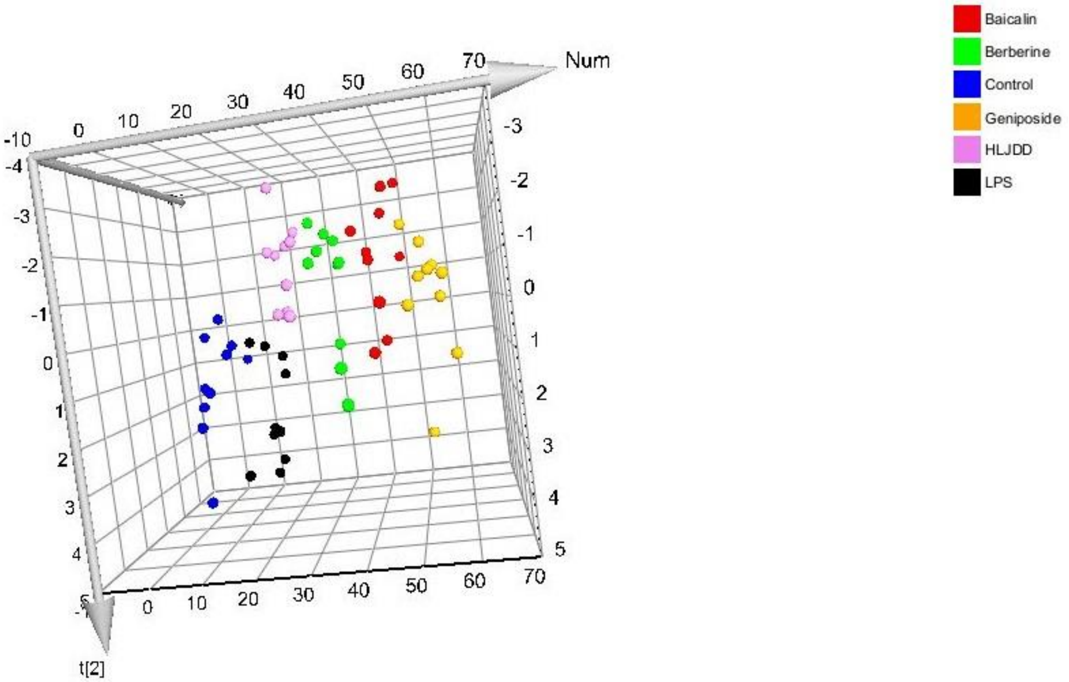

Figure S2. The PCA 3D scores plot of six groups. The colors represented the different groups. The red notes, green notes, blue notes, orange notes, pink notes and black notes represented the baicalin, berberine, control, geniposide, HLJDD and LPS groups, respectively.

Table S1. Statistics of death situation of zebrafish under detection concentration of 4 experiment groups (n = 30)

Group	Concentration（μg/mL）	Mortality（%）	Toxicity
Control	-	0	-
LPS	-	0	-
HLJDD	6.25	0	No obvious toxicity
	12.5	0	No obvious toxicity
	25	0	No obvious toxicity
	50	0	No obvious toxicity
	100	0	No obvious toxicity
Berberine	15.6	0	No obvious toxicity
	31.25	0	No obvious toxicity
	62.5	0	No obvious toxicity
	125	0	No obvious toxicity
	250	0	Heart rate and blood flow slow down
Baicalin	31.25	0	No obvious toxicity
	62.5	0	No obvious toxicity
	125	0	No obvious toxicity
	250	0	No obvious toxicity
	500	20	Obvious toxicity
Geniposide	31.25	0	No obvious toxicity
	62.5	0	No obvious toxicity
	125	0	No obvious toxicity
	250	0	No obvious toxicity
	500	0	No obvious toxicity

Table S2. Identification of potential lipid markers of inflammation differentially expressed in control, LPS, and drug-treatment groups based on non-targeted lipidomics.

No.	Potential lipid markers	t_R/min	m/z	Con vs. LPS			HLJDD vs. LPS		Berberine vs. LPS		Baicalin vs. LPS		Geniposide vs. LPS	
				VIP	P	FC	P	FC	P	FC	P	FC	P	FC
1	PC(22:6/18:3)	10.05	828.5522	1.419	6.25E-05	2.514	9.66E-06	6.220	1.57E-05	4.053	5.22E-04	2.567	1.32E-06	8.845
2	PC(22:6/16:1)	10.37	803.5438	1.181	6.88E-06	2.590	8.88E-06	4.276	6.59E-06	3.469	9.92E-05	2.698	3.13E-07	6.817
3	PC(20:5/20:0)	13.94	835.6073	1.482	3.84E-05	4.290	3.68E-04	6.620	2.75E-04	4.781	6.85E-03	2.671	5.25E-05	13.863
4	PC(20:5/17:1)	13.96	792.5523	3.052	1.59E-04	2.146	2.76E-05	3.043	1.03E-06	3.621	1.15E-02	1.562	1.85E-07	5.238
5	PC(18:4/18:0)	12.18	782.5679	2.051	4.40E-05	2.544	3.48E-05	4.149	8.36E-06	4.028	5.44E-04	2.398	1.10E-06	7.239
6	PC(18:2/20:0)	15.15	814.6297	1.265	4.21E-05	3.782	7.50E-05	5.520	2.46E-05	4.923	3.37E-03	2.473	2.31E-06	20.399
7	PC(22:6/22:6)	10.29	877.5606	1.622	8.62E-04	1.773	1.50E-04	3.128	2.17E-05	3.342	3.80E-02	1.514	2.21E-06	5.703
8	PC(20:5/22:6)	9.87	851.5449	1.326	9.58E-05	1.851	5.93E-06	3.268	8.54E-07	3.338	4.96E-03	1.638	2.20E-08	5.361
9	PC(18:2/22:6)	10.84	829.5607	2.154	4.17E-05	2.678	4.65E-05	4.949	3.52E-05	3.847	1.24E-03	2.520	3.75E-06	8.714
10	PC(18:1/22:6)	11.88	831.5757	6.002	1.29E-04	3.145	5.47E-04	5.697	3.74E-04	4.494	9.81E-03	2.449	7.84E-05	10.978

11	PC(18:1/18:2)	12.59	784.5828	4.784	8.28E-05	3.365	3.48E-04	5.208	2.36E-04	4.114	5.87E-03	2.526	3.71E-05	10.236
12	PC(18:0/22:6)	13.40	833.5917	4.366	6.04E-05	3.382	3.51E-04	4.769	2.21E-04	3.870	1.93E-02	2.006	2.83E-05	9.812
13	PC(18:0/20:5)	12.71	807.5760	3.307	3.45E-05	3.726	1.75E-04	4.807	1.27E-04	3.801	1.17E-02	2.085	1.19E-05	9.852
14	PC(18:0/18:2)	13.90	786.5992	4.944	5.70E-05	3.218	1.63E-04	5.717	1.42E-04	4.158	2.80E-03	2.676	2.26E-05	9.866
15	PC(18:0/18:1)	12.96	810.5988	1.397	4.89E-05	3.081	1.09E-04	6.239	4.23E-05	5.547	9.42E-04	3.128	1.46E-05	10.120
16	PC(16:1/22:6)	10.65	803.5439	1.878	9.28E-05	2.908	6.91E-04	5.208	5.17E-04	4.089	8.88E-03	2.479	1.22E-04	8.343
17	PC(16:1/20:4)	11.22	779.5444	4.742	3.17E-05	2.741	2.42E-05	4.506	1.50E-05	3.782	8.35E-04	2.355	1.36E-06	7.225
18	PC(16:1/18:2)	11.37	755.5450	3.009	1.21E-04	3.168	1.13E-04	6.504	1.27E-04	4.332	1.04E-03	3.158	1.84E-05	10.279
19	PC(16:0/18:2)	12.38	757.5605	4.788	6.11E-05	2.969	1.62E-04	4.756	8.68E-05	4.031	1.96E-03	2.606	1.37E-05	8.670
20	PC(16:0/18:0)	15.01	762.5980	1.497	5.16E-05	2.342	2.05E-05	2.778	6.21E-07	3.081	1.41E-02	1.450	5.14E-08	5.511
21	PC(16:0/16:1)	12.12	731.5451	3.269	2.06E-04	2.497	2.38E-04	4.417	9.80E-05	4.165	3.93E-03	2.297	2.28E-05	7.559
22	PC(16:0/16:0)	13.51	733.5606	3.542	7.11E-04	1.860	1.25E-04	2.414	5.66E-06	2.912	4.99E-02	1.363	7.76E-07	3.965
23	PC(16:0/20:4)	11.52	782.5681	2.992	1.02E-04	3.454	1.62E-04	4.448	1.07E-04	7.945	1.20E-03	3.345	2.38E-05	11.609

24	PC(16:0/18:1)	13.71	759.5762	10.535	1.24E-04	2.877	3.89E-04	4.461	1.74E-04	4.120	8.72E-03	2.176	3.62E-05	8.178
25	PC(O-16:0/20:3)	12.71	792.5877	1.437	1.72E-04	2.664	4.20E-03	2.167	5.18E-04	2.311	6.91E-01	1.091	1.74E-05	4.442
26	PC(15:0/18:2)	13.41	764.5219	2.139	3.62E-04	2.355	2.89E-04	3.620	2.09E-04	2.537	4.05E-02	1.579	2.81E-06	5.371
27	PC(18:3/22:4)	12.00	876.5744	5.211	5.55E-04	2.414	4.74E-05	4.138	1.31E-05	3.300	1.61E-03	2.228	1.39E-06	8.330
28	PC(20:5/20:3)	10.92	874.5584	2.110	1.59E-04	2.377	1.62E-05	3.962	1.49E-05	3.453	1.30E-02	1.898	1.54E-07	9.376
29	PC(20:5/22:4)	11.27	900.5742	1.299	8.37E-04	2.273	3.44E-05	4.999	7.85E-06	4.032	4.44E-02	1.776	6.02E-07	16.186
30	PE(18:0/22:6)	14.10	790.5376	4.132	6.04E-03	1.625	1.54E-03	2.379	5.12E-06	2.562	2.39E-01	1.202	1.72E-07	3.866
31	PE(16:0/22:6)	12.34	762.5063	3.082	4.21E-03	1.648	6.82E-04	2.665	1.26E-06	2.790	5.02E-02	1.354	5.25E-08	4.332
32	PE(18:0/20:4)	14.54	766.5374	1.780	1.15E-03	2.120	4.45E-04	2.581	6.58E-05	2.618	6.57E-03	1.789	9.94E-07	5.582
33	PE(21:0/16:0)	15.09	806.5902	1.533	5.10E-04	1.870	3.72E-04	2.505	1.34E-05	2.337	1.38E-01	1.203	1.03E-07	3.626
34	PE(22:6/18:1)	12.46	788.5219	2.558	3.22E-03	1.685	2.16E-04	3.023	5.58E-07	2.961	3.98E-03	1.629	3.56E-08	4.843
35	PE(P-18:0/20:4)	14.43	767.5450	1.318	2.86E-05	2.747	5.88E-05	3.562	5.57E-06	3.645	9.36E-04	2.157	4.94E-07	7.214
36	PE(22:4/19:0)	13.89	809.5912	1.626	3.32E-05	3.534	1.86E-04	4.898	9.12E-05	4.202	2.63E-03	2.560	1.12E-05	10.991

37	PE(20:5/18:0)	13.27	765.5290	1.552	2.02E-05	2.813	2.85E-05	4.148	1.70E-05	3.371	2.30E-03	2.068	1.20E-06	6.526
38	PE(16:0/22:2)	11.73	794.5686	1.527	3.76E-05	3.462	3.71E-05	6.275	2.51E-05	4.702	1.24E-03	2.590	3.11E-06	12.066
39	PE(18:1/18:0)	15.34	745.5605	1.219	6.94E-05	2.554	1.52E-04	2.879	1.82E-05	2.974	1.26E-02	1.658	1.28E-06	5.799
40	TG(18:3/19:0/19:1)	18.42	910.7964	2.482	3.50E-04	2.973	2.18E-02	1.631	5.92E-04	2.068	4.25E-01	1.188	2.08E-05	3.557
41	TG(17:2/18:0/19:1)	18.38	884.7811	5.749	4.64E-04	2.851	2.84E-02	1.673	3.62E-03	1.858	5.09E-01	1.168	1.02E-04	3.396
42	TG(17:0/18:0/18:3)	18.25	870.7652	2.786	3.31E-04	2.937	1.01E-02	1.909	2.93E-03	1.905	3.67E-01	1.257	1.18E-04	3.329
43	TG(14:0/20:0/20:4)	18.14	882.7654	5.780	3.30E-04	3.053	3.29E-02	1.674	6.02E-03	1.799	4.23E-01	1.221	7.91E-05	3.606
44	TG(14:0/17:1/20:0)	18.49	846.7661	2.339	5.11E-04	2.168	4.57E-02	1.487	1.12E-02	1.591	9.12E-01	0.975	2.43E-03	2.021
45	TG(13:0/19:0/22:2)	18.63	886.7971	3.830	5.11E-04	2.631	3.42E-02	1.525	1.06E-03	1.884	9.64E-01	1.009	6.06E-05	2.952
46	TG(12:0/22:0/22:6)	18.20	924.7996	3.248	4.59E-04	2.879	1.15E-01	1.401	1.87E-03	1.906	7.08E-01	0.918	7.61E-05	3.317
47	TG(18:2/20:0/20:5)	18.20	932.7817	3.254	5.62E-04	2.910	1.36E-01	1.366	8.77E-04	1.988	6.88E-01	0.914	5.46E-05	3.140
48	TG(16:1/19:0/20:3)	18.29	914.8140	1.790	2.76E-04	3.291	6.44E-04	2.328	9.06E-05	2.415	8.37E-02	1.475	5.54E-06	4.547
49	TG(16:0/18:0/20:5)	17.91	880.7484	6.035	4.43E-04	3.018	1.15E-02	1.854	6.66E-03	1.770	4.16E-01	1.225	1.08E-04	3.531

50	SM(d16:1/18:0)	11.97	702.5663	1.332	5.78E-04	1.800	1.50E-03	1.694	3.23E-04	1.741	1.71E-01	1.205	5.75E-05	2.049
51	PA(20:2/21:0)	15.10	788.6136	5.027	3.20E-05	3.139	1.59E-04	3.914	8.81E-05	3.277	1.06E-02	1.945	5.47E-06	8.592
52	PS(18:3/21:0)	13.89	872.5618	2.319	2.12E-04	2.319	1.90E-04	3.511	5.58E-06	2.967	2.89E-03	1.889	9.06E-07	6.267
53	PS(21:0/18:2)	11.86	850.5585	2.238	6.70E-04	2.238	7.34E-05	3.584	1.62E-05	3.017	7.14E-03	1.903	1.56E-06	6.969
54	PS(22:0/15:0)	12.30	826.5589	2.014	6.06E-04	2.014	3.45E-05	3.160	2.15E-05	3.043	1.04E-03	2.003	1.85E-07	5.816
55	PS(22:0/15:1)	11.33	824.5432	2.384	1.86E-04	2.384	6.84E-05	4.063	5.85E-05	3.138	1.33E-03	2.160	6.80E-07	6.527
56	PS(22:6/19:1)	11.33	892.5303	2.846	2.29E-05	2.846	4.25E-05	3.903	1.53E-05	3.655	8.41E-04	2.275	3.68E-07	9.035

Table S3. Identification of potential lipid markers of inflammation differentially expressed in control, LPS, and drug-treatment groups based on targeted lipidomics.

No.	Potential lipid markers	t_R/min	m/z	Con vs. LPS			HLJDD vs. LPS		Berberine vs. LPS		Baicalin vs. LPS		Geniposide vs. LPS	
				VIP	P	FC	P	FC	P	FC	P	FC	P	FC
1	PC(14:0/18:2)	8.59	730.5381	1.436	2.35E-02	1.938	3.40E-04	4.569	2.52E-04	4.841	1.20E-02	2.346	1.96E-04	5.532
2	PC(16:1/16:0)	8.98	732.5538	1.296	3.26E-02	1.827	9.04E-04	3.511	3.31E-04	4.157	3.92E-02	1.873	3.18E-04	4.395
3	PC(20:4/14:0)	8.53	754.5381	1.231	4.79E-02	1.811	1.93E-03	3.417	7.11E-04	4.282	4.06E-02	1.971	3.71E-04	5.443
4	PC(18:3/16:0)	8.83	756.5538	1.436	3.87E-02	1.913	3.13E-04	4.828	4.10E-04	4.328	1.48E-02	2.331	1.13E-04	6.669
5	PC(18:0/16:0)	10.2	762.6007	1.184	2.12E-02	1.671	2.33E-03	2.137	1.15E-04	3.004	2.50E-01	1.276	1.17E-04	3.076
6	PC(P-16:0/20:4)	9.06	766.5745	1.425	3.71E-02	1.863	9.72E-03	2.169	1.11E-03	2.780	4.62E-01	1.219	3.60E-05	0.927
7	PC(18:2/17:0)	9.43	772.5851	1.421	3.44E-02	1.853	2.10E-04	4.245	1.45E-04	4.385	2.02E-02	2.083	4.88E-05	6.114
8	PC(18:1/17:0)	9.91	774.6007	1.386	3.61E-02	1.805	3.86E-04	3.299	1.23E-04	3.842	9.14E-02	1.602	4.19E-05	5.058
9	PC(20:4/16:1)	8.82	780.5538	1.286	4.19E-02	1.756	4.07E-04	3.346	1.59E-04	3.762	6.37E-02	1.729	5.68E-05	4.596
10	PC(18:0/18:2)	9.75	786.6007	1.547	2.32E-02	2.042	4.31E-04	4.317	3.43E-04	4.339	3.24E-02	2.046	1.08E-04	6.618
11	PC(18:0/18:0)	11.09	790.6320	1.189	7.59E-03	1.591	8.62E-04	1.933	8.44E-05	2.284	8.44E-05	2.284	5.62E-05	0.866

12	PC(20:4/18:2)	8.76	806.5694	1.511	2.28E-02	1.991	2.81E-04	4.129	3.00E-04	3.919	6.08E-02	1.897	5.82E-05	6.004
13	PC(20:4/18:1)	9.15	808.5851	1.439	4.50E-02	1.946	1.27E-03	3.980	7.94E-04	4.325	4.48E-02	2.016	3.66E-04	5.611
14	PE(0:0/22:6)	4.79	526.2928	1.303	2.25E-02	1.866	4.53E-03	2.266	9.70E-04	3.002	7.14E-01	1.108	1.16E-04	0.673
15	PE(18:2/16:0)	9.16	716.5225	1.291	1.80E-02	1.718	1.38E-04	3.302	8.90E-06	4.216	7.00E-03	1.931	1.51E-05	3.972
16	PE(18:2/18:0)	9.79	744.5538	1.463	2.80E-02	1.972	3.59E-04	4.909	2.95E-04	4.962	1.12E-02	2.481	1.40E-04	6.705
17	PE(18:0/18:1)	9.32	746.5694	1.286	3.25E-02	1.791	6.21E-04	3.452	1.76E-04	4.174	3.67E-02	1.850	1.06E-04	4.835
18	PE(22:6/16:0)	8.97	764.5225	1.073	4.37E-02	1.573	6.85E-04	2.613	3.94E-05	3.700	6.00E-02	1.532	6.43E-05	3.485
19	PE(20:4/18:0)	9.67	768.5538	1.425	2.53E-02	1.818	4.72E-04	2.907	8.32E-05	3.557	1.62E-02	1.855	3.60E-05	4.124
20	PE(18:0/22:6)	9.54	792.5538	1.137	3.43E-02	1.620	6.70E-04	2.544	3.42E-05	3.658	1.18E-01	1.413	5.62E-05	3.389
21	Cer(d18:1/16:0)	11.91	540.5350	1.190	2.32E-02	1.672	2.02E-02	1.604	2.43E-03	2.186	8.24E-01	0.948	9.97E-01	0.906
22	Cer(d18:1/22:0)	11.68	624.6289	1.234	4.81E-03	1.603	7.19E-02	1.293	4.57E-03	1.626	6.67E-01	0.922	2.84E-01	1.208
23	Cer(d18:1/23:0)	12.3	636.6289	1.177	1.66E-02	1.580	2.70E-01	1.180	7.03E-03	1.648	3.47E-01	0.780	3.35E-01	1.183
24	Cer(d18:0/24:1)	12.22	650.6446	1.046	1.57E-02	1.439	1.61E-01	1.190	7.87E-03	1.502	6.11E-01	0.907	6.87E-01	1.142

25	SM(d18:1/22:0)	11.68	787.6688	1.014	4.43E-02	1.404	1.20E-01	1.220	2.23E-03	1.511	9.59E-01	0.992	7.19E-05	0.988
